# Molecular Pathology, Artificial Intelligence, and New Technologies in Hematologic Diagnostics: Translational Opportunities and Practical Considerations

**DOI:** 10.3390/diagnostics16060913

**Published:** 2026-03-19

**Authors:** Fnu Alnoor, Shuvam Mukherjee, Madhu P. Menon, David Ng, Peng Li, Robert S. Ohgami

**Affiliations:** 1 Department of Pathology and Laboratory Medicine, Division of Hematopathology, University of Miami Miller School of Medicine, Miami, FL 33136, USA; 2 Department of Computer Engineering, University of Maryland, College Park, MD 20742, USA; 3 Department of Pathology, University of Utah School of Medicine, Salt Lake City, UT 84112, USArobert.ohgami@aruplab.com (R.S.O.); 4 ARUP Laboratories, Division of Hematopathology, Salt Lake City, UT 84108, USA; 5 ARUP Laboratories, Institute for Research and Innovation in Diagnostic and Precision Medicine, Salt Lake City, UT 84108, USA

**Keywords:** hematopathology, molecular pathology, automation, artificial intelligence

## Abstract

**Background and Objectives:** Diagnostics for hematologic diseases rely on integrated assessment of clinical manifestation, morphology, flow cytometry, and molecular testing. Current classification systems, including the WHO HAEM5 and the International Consensus Classification, highlight the central role of genomics in defining disease entities and risk. Simultaneously, laboratories face growing case complexity and staffing challenges. Automation, collaborative robots (cobots), digital morphology platforms, and artificial intelligence (AI) have begun to address these issues. Here we examine the application of these technologies in hematopathology and molecular diagnostics and consider their translational potential to improve diagnostic accuracy and, ultimately, patient care. **Methods:** A review of peer-reviewed literature and technical reports published through December 2025 was performed, focusing on digital morphology platforms, AI for peripheral blood and marrow interpretation, AI-enabled flow cytometry, automated and robotic deployments in clinical laboratories, and machine learning applications in molecular hematopathology. **Results:** Digital morphology analyzers show strong concordance with manual microscopy and now serve as efficient platforms for AI-assisted differentials, cell classification, and fibrosis quantification. Deep learning applied to multiparameter flow cytometry achieves performance comparable to expert review in distinguishing mature B-cell neoplasms and acute leukemias. Automated solutions, cobot systems and robotic-arm-based slide-scanning clusters have demonstrated substantial gains in throughput and pre-analytic consistency. AI models in molecular hematopathology increasingly assist with variant interpretation, genetic risk stratification, and linking morphologic and genomic findings. **Conclusions:** AI is beginning to change how hematopathology and molecular diagnostics are practiced. Successful translation will depend on disease-specific validation, the development of multi-modal models aligned with ICC and WHO frameworks, and laboratory governance that maintains expert oversight.

## 1. Introduction

Hematopathology and molecular hematology now rely on workflows that routinely integrate peripheral blood and bone marrow morphology, complex flow cytometry panels, and targeted sequencing. The 5th edition of the World Health Organization (WHO) classification and the International Consensus Classification (ICC) for myeloid neoplasms and acute leukemias have relied heavily on recurrent genomic abnormalities to assign diagnostic criteria and risk groups, underscoring the central role of molecular pathology in this field [1,2]. What this ultimately means is that pathologists must synthesize larger volumes of heterogeneous data under tighter time constraints than in the past.

Meanwhile, the physical processes of specimen handling in many laboratories have changed little over the years. Technologists still prepare smears, operate microtomes, load and unload analyzers, and carry racks of tubes and slides between benches. Pathologists still spend substantial time at microscopes reviewing smears, marrow aspirates, and core biopsies, field by field. Such a model is increasingly difficult to sustain given rising diagnostic complexity and persistent staffing constraints.

Interestingly, core and hematology laboratories faced these pressures earlier and adopted total laboratory automation (TLA) [3]. In anatomic pathology, the transition is taking a different form. Automation and cobots can absorb repetitive pre-analytical and analytical tasks. Digital morphology provides stable, shareable image data. AI can act on these digital inputs and on structured flow cytometry and genomic data to support triage, quantification, and, eventually, multi-modal reasoning.

The key question now is how to deploy these technologies in ways that are clinically meaningful, compatible with ICC and WHO frameworks, and realistic for laboratories facing budget and staffing constraints.

Our review is intended for practicing hematopathologists, molecular pathologists, laboratory directors, and translational researchers evaluating whether and how AI-enabled technologies can be integrated into routine diagnostic workflows.

## 2. Methods

Peer-reviewed publications and technical papers through December 2025 were identified using PubMed, Google Scholar, and online searches. The search focused on work involving digital morphology platforms for peripheral blood, bone marrow, and tissue imaging; deep learning applied to hematologic morphology; artificial intelligence in flow cytometry; automation and collaborative robotics in clinical laboratories; and machine learning approaches in molecular hematopathology. Economic and financial evaluations of digital pathology and laboratory automation were also reviewed when they provided specific data relevant to implementation.

## 3. Results

### 3.1. Machine Learning Paradigms in Hematologic Diagnostics

AI applications in hematologic diagnostics employ a spectrum of learning paradigms. Supervised learning dominates morphology and flow cytometry applications, where expert-labeled datasets enable models to learn cell classification and disease-specific patterns. Unsupervised and semi-supervised approaches are increasingly applied to high-dimensional flow cytometry and genomic datasets, facilitating identification of rare populations, clonal structure, and disease heterogeneity. Deep learning methods, particularly convolutional neural networks and transformer-based architectures, are widely used for image-based analysis and emerging multimodal integration tasks across morphology, flow cytometry, and molecular data [4,5,6,7].

### 3.2. Automation and Collaborative Robotics in Hematology and Molecular Pathology

At the most basic level, many laboratories begin automation efforts with simple liquid handling systems and benchtop automation rather than full robotic workcells. Automated pipettors and compact liquid handlers are commonly used for repetitive steps such as aliquoting, reagent addition, PCR setup, and plate normalization in molecular and hematology workflows. These systems are relatively low cost, require minimal floor space, and can often be programmed by laboratory staff without specialized engineering support. By improving precision and reproducibility while reducing hands-on time and technologist fatigue, simple liquid handling automation provides a practical on-ramp to more advanced robotic and AI-enabled laboratory workflows.

Collaborative robots (Cobots) are designed to work in proximity to humans, combining torque-limited joints and safety features with relatively simple programming, and have been implemented for repetitive, structured tasks such as tube sorting, slide loading, and plate handling that are common across clinical laboratories (Table 1). For example, Copenhagen University Hospital in Gentofte has used two UR5 cobots to handle and sort blood samples for analysis. These cobots pick up tubes from a conveyor, position them for barcode reading and cap color detection, distribute them into racks, and load racks into analyzers. The system handles approximately 3000 samples per day and enables the laboratory to maintain its target of delivering over 90% of results within one hour, despite a substantial increase in volume, without adding staff [8]. In this setting, the cobots have relieved technologists from repetitive, ergonomically challenging tasks and allowed them to focus on more complex work.

Similar concepts are being applied in digital pathology. Pramana’s Spectral HT series is an example of a high-throughput whole-slide imaging platform that uses a robotic arm as an integral part of the design. A typical cluster consists of four single-slide line scanners arranged around a central robotic handler that feeds slides from a shared tray into any of the scanners. Each scanner operates independently, while the robotic arm continuously loads, unloads, and reallocates slides based on scanning status and quality metrics. In a high-volume environment, a single cluster can scan over 1000 slides per day, with one operator overseeing the process [9]. At Caris Life Sciences, a Spectral HT system is part of a scanning lab that processes ~1.5 million slides per year; in one validation study of 867 slides, automated quality control from the Pramana system achieved a 100% scan success rate and showed >98% agreement with manual quality review. At Vanderbilt University Medical Center, a Pramana system with four scanning compartments surrounding a central robotic arm has been deployed to digitize roughly 900 slides per day, with a planned total of 500,000 slides over three years [10]. These robotic-arm-based scanning clusters can be considered cobot systems, in that they occupy the same general workspace as technologists, can be interrupted or adjusted as needed, and are designed to be reconfigurable and integrated into workflows in ways that combine high throughput with human oversight.

For hematology and hematopathology, the relevance is clear. These same robotic concepts can be applied to EDTA tube handling, smear preparation, slide loading for digital morphology, and plate loading for high-throughput flow cytometry and molecular or cytogenetic assays. Cobots and robotic-arm-based scanners can provide a stable physical foundation for AI platforms to build on.

While automation and collaborative robotics primarily address pre-analytic and physical workflow challenges, digital morphology represents the next step by transforming glass slides into standardized image data that enables downstream AI-assisted interpretation.

**Table 1:** Examples of automation and robotic-arm/cobot applications relevant to hematology and digital pathology.

Application	Platform/Site	Domain	Key Points
Pre-analytic blood tube handling	UR5 cobots at Copenhagen University Hospital Gentofte	Core hematology	Two UR5 arms sort and load tubes into analyzers, processing ~3000 samples/day while maintaining a target of >90% results within one hour without additional staff.
High-throughput slide scanning cluster	Pramana Spectral HT cluster with robotic arm	Digital pathology	Four single-slide scanners around a central robotic handler; one cluster can scan >1000 slides/day with automated quality control and real-time error detection.
Large-scale precision medicine operations	Caris Life Sciences and Pramana partnership	Digital oncology	Caris integrates Pramana Spectral HT in a multi-scanner lab processing ~1.5 million slides/year; reported 100% scan success in a study of 867 slides with >98% agreement between automated and manual QC.
Academic pathology digitization	Vanderbilt University Medical Center and Pramana	Research and clinical digitization	Pramana system with four scanning compartments and a central robotic arm digitizes ~900 slides/day; pilot aims to scan 500,000 slides over three years for research and planning for primary digital diagnosis.
Hematopathology-specific AI collaboration	ARUP-Pramana partnership	Hematopathology and AI	Collaboration uses HT scanners and hematopathology expertise to develop AI algorithms for bone marrow biopsies and other hematopathology challenges, with an emphasis on edge AI deployment.

### 3.3. Digital Morphology and AI in Peripheral Blood and Bone Marrow

Digital morphology systems form the bridge between pre-analytic automation and AI-based interpretation (Table 2). CellaVision DM-series analyzers and Scopio full-field platforms are now widely used in hematology laboratories and have been evaluated in multiple studies. In fact, one evaluation of the CellaVision DM96 system showed high concordance between automated differentials and manual microscopy for most white blood cell categories, including blasts, and suggested that the system could safely reduce manual smear review for routine cases [11]. Other studies have confirmed that CellaVision systems improve efficiency and support remote review and consultation in routine hematology practice [11,12]. Scopio’s X100 platform introduced full-field imaging of peripheral blood smears, capturing the entire smear at high resolution, including the feathered edge. In an evaluation that included both normal and abnormal smears, including smears with blasts and other significant abnormalities, Scopio-based differentials showed high correlation with manual counts and reduced review time, especially when AI-based pre-classification was used as an initial sorting step [12]. A later study of the X100HT system confirmed these findings and reinforced the practical utility of AI to support routine pathology practices [13]. Reported concordance between digital morphology analyzers and manual microscopy varies by platform, study design, and cell category, but typically ranges from approximately 85% to 98% for major leukocyte populations. Studies evaluating CellaVision and Scopio platforms often involve cohorts ranging from ~100 to more than 1000 peripheral blood smears. In most validation studies, manual differentials are performed by experienced laboratory technologists and/or hematopathologists, frequently with consensus adjudication in discrepant cases. Importantly, AI-assisted preclassification generally improves both concordance and review efficiency by prioritizing abnormal cells for expert confirmation rather than replacing manual interpretation [11,12,13]. Beyond diagnostic concordance, AI-enabled digital morphology provides important workflow advantages in hematology laboratories facing increasing case complexity and strict turnaround expectations. AI-assisted preclassification has been shown to improve review efficiency and may enhance concordance by prioritizing abnormal cells for expert confirmation, enabling pathologists and technologists to focus on diagnostically significant findings rather than routine enumeration. These systems also facilitate remote consultation, standardized quantification of morphologic features, and improved reproducibility across observers. Collectively, these benefits contribute to reduced review time, improved workflow efficiency, and better management of high smear volumes without replacing expert interpretation [14].

Full-field digital bone marrow aspirate and biopsy imaging is less mature, but already significant advancements have been made. Early deployments of full-field marrow imaging systems have demonstrated that aspirate smears and trephine biopsies can be digitized at a resolution suitable for detailed review and AI analysis [15]. A recent collaboration between ARUP Laboratories and Pramana specifically targeted bone marrow biopsies for AI development using Pramana’s Spectral HT scanners and ARUP’s hematopathology expertise [16]. These collaborations will be important as the field moves from peripheral blood morphology to more complex marrow- and tissue-based hematopathology.

Deep learning applied to digital hematologic morphology has matured from proof-of-concept toward task-specific clinical utility, particularly in areas where reproducibility and quantification are challenging for human observers. Convolutional neural networks trained on large cell-image datasets have demonstrated excellent performance in blast identification, lineage assignment, and multi-class marrow cell classification, supporting their use as screening and quantification tools rather than independent diagnostic arbiters.

These systems show their greatest value in standardizing routine but labor-intensive tasks prone to significant interindividual variability, such as differential counts, fibrosis quantification, and cellularity assessment, while leaving contextual interpretation and diagnostic synthesis to hematopathologists. As with any morphology-based tool, disease-specific validation and stain-aware and platform-aware performance monitoring remain essential for safe clinical deployment. For instance, a simple convolutional neural network trained on tens of thousands of single-cell images achieved human-level performance in recognizing blast cells in AML [4]. Other models trained on microscope images of peripheral blood and marrow have been shown to bin common leukemia subtypes with high accuracy [17]. A more recent ensemble model, trained on tens of thousands of cell images from marrow aspirates, achieved high F1 scores across more than 20 cell classes and demonstrated strong performance on an external cohort [18]. Additionally, in bone marrow biopsies, machine learning-based quantification of reticulin fibrosis has been shown to provide more reproducible and more granular fibrosis indices than conventional semi-quantitative grading and to correlate with clinical outcomes [19]. AI-based quantitation of marrow cellularity and dysplasia in myelodysplastic syndromes has also been reported [20].

These results suggest that once digital morphology is in place, AI will complete much of the routine counting and screening work in hematopathology. Cobots and robotic-arm-based scanners can ensure that the slides arriving at these digital platforms are prepared and delivered consistently. Similar principles of digital transformation apply to flow cytometry, where high-dimensional structured data provide an additional substrate for AI-based classification and diagnostic decision support.

### 3.4. AI in Flow Cytometry

Flow cytometry is central to hematologic diagnosis and is an obvious target for AI, as it produces structured, multidimensional data. Recent work has demonstrated that deep learning applied to routine multiparameter flow data can achieve diagnostic performance comparable to expert interpretation (Table 2).

In one recent study of mature B-cell neoplasms, a deep learning model trained on multiparameter flow cytometry data classified cases at a level that matched or exceeded pathologists’ review. The model ingested full event-level data, rather than manually gated populations, and was able to distinguish between several subtypes of B-cell disorders [21]. Another study applied AI-assisted analysis to flow cytometry of acute leukemia and achieved high sensitivity and specificity for distinguishing acute leukemia from non-leukemic samples, while also providing lineage assignments that matched expert diagnoses [22]. Recognizing the need for structured guidance, an expert panel published recommendations for the use of AI in clinical flow cytometry. These recommendations addressed data standardization, model validation and revalidation, performance monitoring, integration with laboratory information systems, and documentation of limitations [5]. A broader review described supervised and unsupervised machine learning methods for clinical flow cytometry and highlighted issues such as domain shift and the need for explainable outputs [6]. Reviews focusing on measurable residual disease in AML and other hematologic malignancies have emphasized that AI-based MRD tools need to be evaluated not only in terms of sensitivity and specificity but also in terms of how they perform in the context of longitudinal patient care [23,24].

Cobots offer an opportunity to relieve some of the pre-analytic workload in flow cytometry. A cobot positioned in front of a flow cytometer could label tubes, vortex samples, and load racks, allowing technologists to supervise overall operations rather than performing each step manually. In the context of high-throughput laboratories with multiple flow cytometers, or laboratories that run large leukemia and lymphoma panels, this would free human staff to focus on assay design, troubleshooting, and interpretation. Beyond morphology and immunophenotyping, molecular testing generates complex genomic datasets that expand AI’s role from pattern recognition to multimodal integration and prognostic modeling.

### 3.5. AI in Molecular Pathology of Hematologic Neoplasms

Molecular pathology is now integral to the diagnosis, classification, and risk stratification of hematologic neoplasms, with the ICC and WHO frameworks explicitly incorporating recurrent genetic alterations into disease definitions. This centrality of genomics, combined with increasing assay complexity and expectations for rapid turnaround, creates a natural role for artificial intelligence as a scaling and integration tool rather than an autonomous diagnostic system. Multimodal testing at diagnosis and longitudinally during treatment offers opportunities to integrate AI into individual testing modalities as well as routine laboratory workflows. AI-assisted karyotype analysis has been shown to improve diagnostic sensitivity and consistency [25], and AI-based approaches have similarly enhanced the efficiency and robustness of NGS data processing and analysis (Table 2).

In the clinical setting, accurate identification and interpretation of clinically significant genetic variants are essential for diagnostic classification and therapeutic decision-making. Within routine NGS workflows, AI-enabled approaches have primarily focused on optimizing discrete analytical steps, including variant calling, annotation, prioritization, and pathogenicity assessment [7,26]. These tools improve consistency and efficiency, particularly in high-throughput laboratories, but remain tightly coupled to expert molecular pathologist review for final clinical interpretation. In molecular hematopathology, AI-based tools improve workflow efficiency by prioritizing clinically relevant variants, identifying complex mutational constellations, and aligning genomic findings with WHO and ICC classification frameworks. These approaches facilitate more efficient interpretation of large sequencing datasets while preserving the central role of expert molecular pathologist review.

Acute leukemias represent clinical emergencies that require rapid diagnostic evaluation; however, current multimodal testing paradigms often generate fragmented diagnostic information over several days, which may delay initiation of optimal therapy. Recent advances in long-read sequencing technologies have enabled a more comprehensive characterization of disease biology, extending beyond conventional genetic abnormalities to include disease-associated epigenomic features. Leveraging these advances in genomics and AI, Steinicke et al. developed MARLIN, a neural network-based model for rapid epigenomic classification of acute leukemia. In their study, the model accurately classified all evaluated cases within a two-hour turnaround time [27]. These approaches highlight the potential for AI to reduce diagnostic latency arising from staggered assay turnaround times, particularly in acute leukemia.

Molecular MRD assessment has become a critical component of diagnosis, risk stratification, and response monitoring in hematologic neoplasms, particularly acute leukemias and other myeloid malignancies [28,29]. NGS-based MRD assays enable sensitive detection of residual disease through tracking of patient-specific somatic variants, but they also generate complex, high-dimensional data across multiple loci and longitudinal time points. AI approaches are increasingly being explored to support molecular MRD workflows by assisting with variant prioritization, distinguishing true residual disease from technical noise, and modeling clonal dynamics over time, rather than making autonomous MRD determinations. Expert consensus guidelines emphasize that molecular MRD interpretation must be performed longitudinally and in a clinical context, as isolated low-level variants may reflect clonal hematopoiesis, therapy-related selection, or assay artifacts rather than persistent disease. AI-based methods can assist with variant prioritization and modeling of clonal dynamics but do not replace expert clinical interpretation [7,25,26,28,29,30]. As molecular MRD assays expand to include broader genomic and epigenomic features, AI-based methods may help manage analytic complexity and reduce diagnostic latency, while final interpretation and clinical integration remain the responsibility of molecular pathologists [7,26,30].

A complementary, increasingly explored strategy involves integrating multimodal data to develop disease-specific risk-stratification and prognostic models. Several groups have applied machine learning to large cohorts of patients with MDS and AML, combining mutational profiles, cytogenetic data, and clinical variables to improve prognostic performance compared with traditional scoring systems [31,32]. While many of these models rely on methods such as gradient boosting or regularized regression rather than deep learning, they share a common objective of capturing complex, multivariable relationships that are difficult to model using conventional approaches.

Although many early AI studies have focused on acute leukemias and myeloid neoplasms, similar approaches are increasingly being explored across a broader spectrum of hematologic diseases. In plasma cell neoplasms such as multiple myeloma, AI-based models are being developed to assess marrow infiltration patterns, integrate cytogenetic and genomic risk features, and support prognostic stratification. Likewise, in lymphoid malignancies, machine learning methods are being applied to immunophenotypic classification, mutation profiling, and outcome prediction. These developments highlight the broader applicability of AI-assisted multimodal analysis across hematologic diagnostics beyond leukemia-focused workflows [33].

There is also a growing body of work integrating morphological and genomic data using AI-based methods. Studies examining image-based prediction of molecular alterations, including *IDH1/2*, *NPM1*, and *FLT3* mutations in AML, as well as marrow histopathology in myelodysplastic syndromes, suggest that certain morphologic patterns may correlate with underlying genetic alterations and risk categories, even when such associations are not readily apparent by conventional morphologic assessment [32,34, 35, 36].

In parallel, collaborative robots (cobots) in molecular laboratories can support upstream processes required for high-quality sequencing and mutational analysis, including automated liquid handling and plate-based library preparation. Downstream, AI-based systems can assist with data interpretation by prioritizing variants, identifying complex or unexpected combinations of genetic lesions, and facilitating alignment with WHO and ICC classification frameworks. Nonetheless, hematopathologists and molecular pathologists remain essential for the final integrated interpretation and diagnostic determination.

### 3.6. Translational and Economic Considerations

Economic analyses from digital pathology and laboratory automation provide a foundation for understanding AI and robotics in hematology. One projection from a large integrated health system has shown that full digital pathology implementation could save $18 million dollars over five years, largely through reduced diagnostic errors and improved efficiency [25,37]. And a study of eight European laboratories implementing digital pathology found that investments reached positive net present value within seven years and became cash flow positive after roughly three years, driven by increased productivity and reduced slide-handling costs [37]. A cost analysis from an anatomic pathology laboratory in Malaysia showed that salaries accounted for more than forty percent of total expenditures, underscoring the potential economic impact of automation in labor-intensive areas [38].

Hematology and hematopathology are particularly sensitive to improvements in pre-analytic consistency and throughput. Smear volumes can be high, bone marrow and lymph node cases often involve many slides, and flow cytometry and molecular tests add additional work. Even incremental improvements in smear preparation, digital morphology triage, or flow cytometry setup can translate into measurable changes in turnaround time and labor needs. When those improvements are linked to AI that triages cases (for example, highlighting likely acute leukemia or clearly reactive patterns) and to automation and cobots that absorb manual handling, the cumulative effect could be substantial.

**Table 2:** Representative AI applications in hematologic morphology, flow cytometry, and molecular pathology.

Domain	Representative Work	Use Case	Relevance
Peripheral blood and marrow smears	Matek 2019 [4]; Ahmed 2019 [17]; Goldgof 2023 [18]	Blast detection; leukemia subtype identification; marrow cell classification	Demonstrated that CNNs can reach human-level performance in blast recognition and assign leukemia subtypes from digital smears and marrow images; ensemble models can classify >20 marrow cell classes.
Marrow biopsies and fibrosis	Ryou 2023 [19]; Yu 2020 [20]	Quantitative fibrosis grading; MDS pattern analysis	ML-based fibrosis indices improve reproducibility and granularity; AI-supported histologic analysis aids in diagnosing MDS and linking morphology to genetic profiles.
Flow cytometry	Zhao 2020 [21]; Zhong 2022 [22]; Ng 2024 [5]; Spies 2025 [6]	Classification of B-cell neoplasms; acute leukemia diagnosis; AI implementation guidance	Deep learning on multiparameter flow data achieves hematologist-level classification; AI-assisted algorithms support acute leukemia diagnosis; expert frameworks guide AI deployment in flow cytometry.
MRD and longitudinal monitoring	Mocking 2025 [23]; Fuda 2023 [24]	MRD assessment in AML and other neoplasms	ML methods help standardize immunophenotypic MRD assessment and support risk-adapted treatment decisions.
Molecular risk models in myeloid neoplasms	Nazha 2021 [31]; Awada 2021 [32]; Al-Nusair 2025 [30]	Prognostic modeling in MDS and AML	Machine learning-based risk scores combine mutations, cytogenetics, and clinical data to refine prognosis and complement ICC- and WHO-based frameworks.
Morphology-genomics linkage	Kockwelp 2023 [35]; Yu 2020 [20]	Image-based prediction of mutations; morphology-mutation correlation	Image-based models predict therapy-relevant mutations in AML and link marrow histology to specific mutational profiles, suggesting that AI can bridge micro- and genomic scales.

## 4. Conclusions

Hematopathology and molecular pathology are entering a phase where automation, cobots, digital morphology, and AI are no longer experimental. Cobots can absorb repetitive physical work in pre-analytic and analytic phases while leaving interpretive and integrative tasks to hematopathologists and molecular pathologists. Digital morphology platforms such as CellaVision and Scopio, along with autonomous robotic-arm-based scanning clusters like Pramana’s Spectral HT systems, provide the image data needed for AI and facilitate large-scale digitization efforts. AI tools for morphology, flow cytometry, and genomics are reaching a level of maturity that justifies serious consideration for routine use.

**Figure 1:**
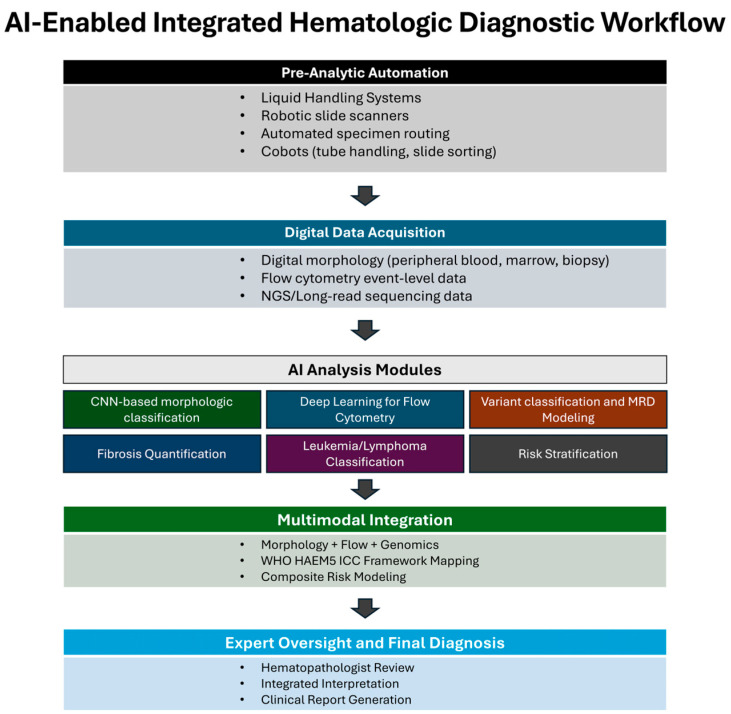
Conceptual overview of an AI-enabled integrated hematologic diagnostic workflow. Pre-analytic automation systems provide standardized specimen handling and slide preparation. Digital platforms convert morphology, flow cytometry, and genomic assays into structured data. AI modules perform task-specific analyses within each modality, followed by multimodal integration aligned with WHO and ICC frameworks. Final interpretation and clinical reporting remain under expert hematopathologist oversight.

The translational task now is to design disease- and modality-specific validation studies, economic evaluations, and governance structures that align with the ICC and WHO frameworks and support patient-centered care. If that work is done carefully and led by domain experts, AI and robotics can become integral parts of a modern, multi-modal diagnostic practice in hematology rather than being imposed on it as external technologies. A conceptual overview of an integrated workflow is shown in Figure 1.

## Data Availability

No new data were created or analyzed in this study.
